# Supplementary material for: Extracellular Matrix Sulfation in the Tumor Microenvironment Stimulates Cancer Stemness and Invasiveness
Source: Adv Sci (Weinh). 2024 Jul 31;11(36):2309966. doi: 10.1002/advs.202309966 (PMC11423251; doi:10.1002/advs.202309966)
Supplement: Supplementary file 1 — Supporting Information [file ADVS-11-2309966-s001.pdf]

ADVANCED SCIENCE
Open Access

Supporting Information

for *Adv. Sci.*, DOI 10.1002/advs.202309966

Extracellular Matrix Sulfation in the Tumor Microenvironment Stimulates Cancer Stemness and Invasiveness

*Alican Kuşoğlu, Deniz Örnek, Aslı Dansık, Ceren Uzun, Sena Nur Özkan, Sevgi Sarıca, Kardelen Yangın, Şevval Özdinç, Duygu Turan Sorhun, Nuriye Solcan, Efe Can Doğanalp, Øystein Arlov, Katherine Cunningham, Ismail C. Karaoğlu, Seda Kizilel, Ihsan Solaroğlu, Pınar Bulutay, Pınar Fırat, Suat Erus, Serhan Tanju, Şükrü Dilege, Gordana Vunjak-Novakovic, Nurcan Tuncbag and Ece Öztürk**

SUPPLEMENTARY INFORMATION

Extracellular matrix sulfation in the tumor microenvironment stimulates cancer stemness and invasiveness

Alican Kuşoğlu[1,2,3 #], Deniz Örnek[1,2,3 #], Aslı Dansık[1,2,4], Ceren Uzun[4], Sena Nur Özkan[1,2,3], Sevgi Sarıca[1,2,3], Kardelen Yangın[1,2,3], Şevval Özdinç[1,2,3], Duygu Turan Sorhun[1,2,3], Nuriye Solcan[1,2,3], Efe Can Doğanalp[1,2], Øystein Arlov[5], Katherine Cunningham[6], İsmail C. Karaoğlu[7], Seda Kizilel[2,7], Ihsan Solaroglu[2,8], Pınar Bulutay[9], Pınar Fırat[9], Suat Erus[10], Serhan Tanju[10], Şükrü Dilege[10], Gordana Vunjak-Novakovic[6], Nurcan Tuncbag[2,7,11], Ece Öztürk[1,2,11]*

[1] Engineered Cancer and Organ Models Laboratory, Koç University, Istanbul 34450, Turkey

[2] Research Center for Translational Medicine (KUTTAM), Koç University, Istanbul 34450, Turkey

[3] Graduate School of Health Sciences, Koç University, Istanbul 34450, Turkey

[4] Graduate School of Sciences and Engineering, Koç University, Istanbul 34450, Turkey

[5] Department of Biotechnology and Nanomedicine, SINTEF Industry, Trondheim 7034, Norway

[6] Department of Biomedical Engineering, Columbia University, NY10032, USA

[7] Chemical and Biological Engineering, Koç University, Istanbul 34450, Turkey

[8] Department of Neurosurgery, School of Medicine, Koç University, Istanbul 34450, Turkey

[9] Department of Pathology, School of Medicine, Koç University, Istanbul 34450, Turkey

[10] Department of Thoracic Surgery, School of Medicine, Koç University, Istanbul 34450, Turkey

[11] Department of Medical Biology, School of Medicine, Koç University, Istanbul 34450, Turkey

# These authors contributed equally.

* Corresponding author

Supplementary Figures

Figure S1

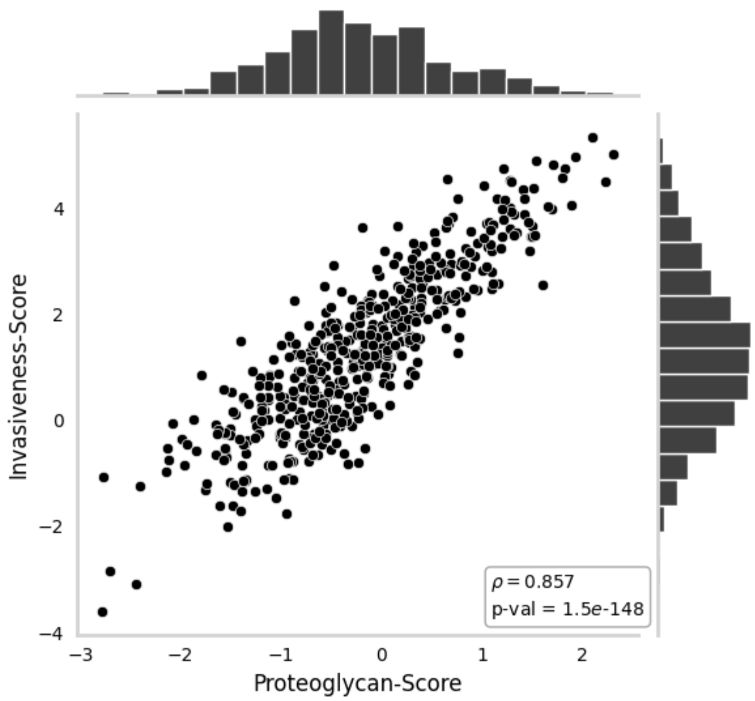

Figure S1. Elevated proteoglycan expression correlates with invasiveness. Regression analysis on invasiveness scores and proteoglycan scores using the mRNA ($p = 1.5e-148$). Spearman rank correlation was used to calculate the correlation value $\rho = 0.86$.

Figure S2

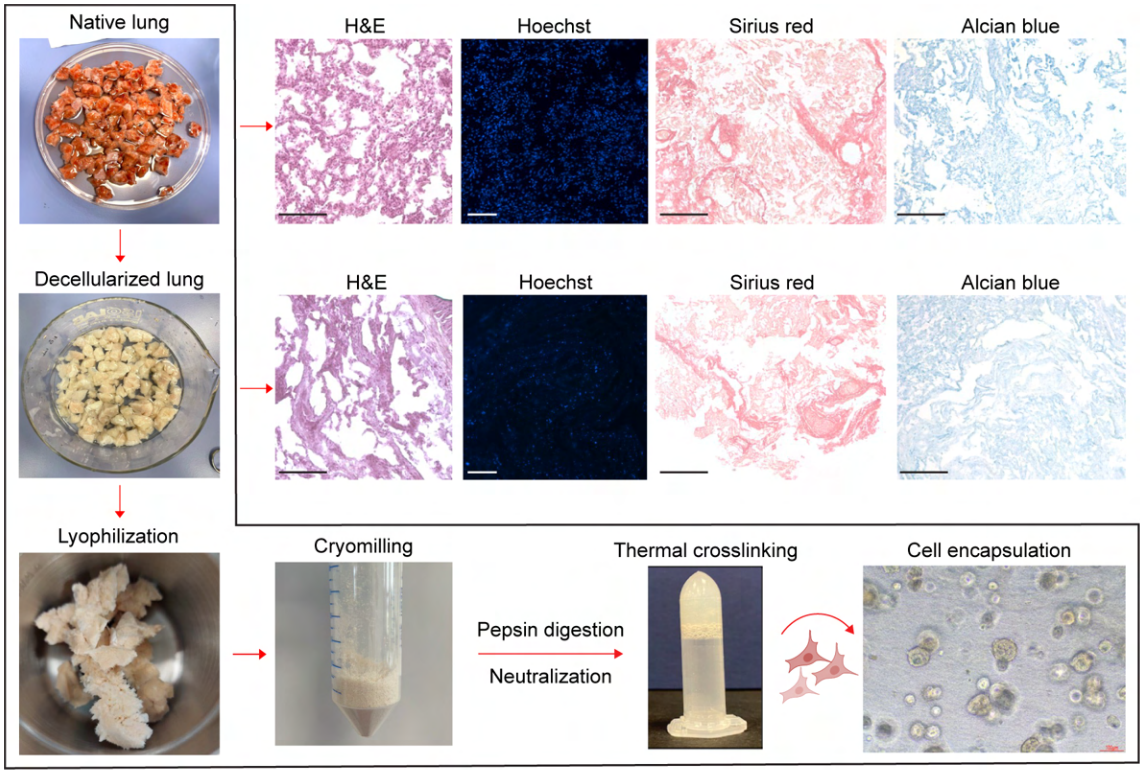

Figure S2. Steps of bovine lung decellularization and characterization of decellularized tissue. Native bovine lung was cut into small pieces, washed throughly with ddH$_2$O supplemented with 1% Penicillin/Streptomycin/Amphotericin (P/S/A) followed by freeze-thaw cycles in liquid nitrogen. Decellularized lung tissue pieces were lyophilized, cryomilled into a powder form and digested in pepsin solution (1 mg/ml in 0.01 M HCl, pH:2) for 48 hours at room temperature. After pepsin digestion, pre-gel solutions were neutralized using NaOH and buffered to neutral pH. A549 cells were encapsulated into dLung hydrogels and monitored for cellular growth via brightfield microscopy. Elimination of nuclear content in decellularized lung tissues was shown by Haematoxylin & Eosin as well as Hoechst stainings in comparison to native tissue. Collagen and sGAG retention in decellularized lung tissues were revealed by Sirius Red and Alcian Blue stainings, respectively (Scale bar: 100 μm).

Figure S3

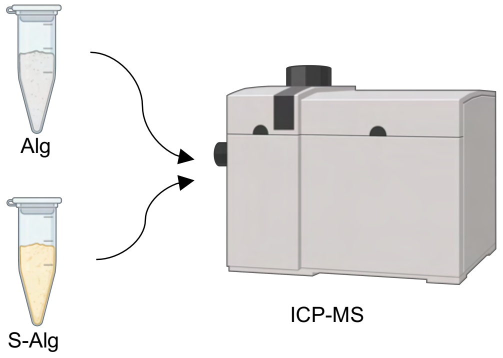

Sample	S		Degree of Sulfation (DS)
	mg/kg	RSD (%)	Sulfate groups/monomer
Alg	621	3,2	0
S-Alg	46 698	2,3	0.41

Figure S3. Analysis of sulfate content in S-Alg compared to Alg. Elemental analyses were performed with high-resolution inductively coupled plasma mass spectrometry (ICP-MS) for determining sulfur content in Alg and S-Alg samples (RSD: Relative Standard Deviation).

Figure S4

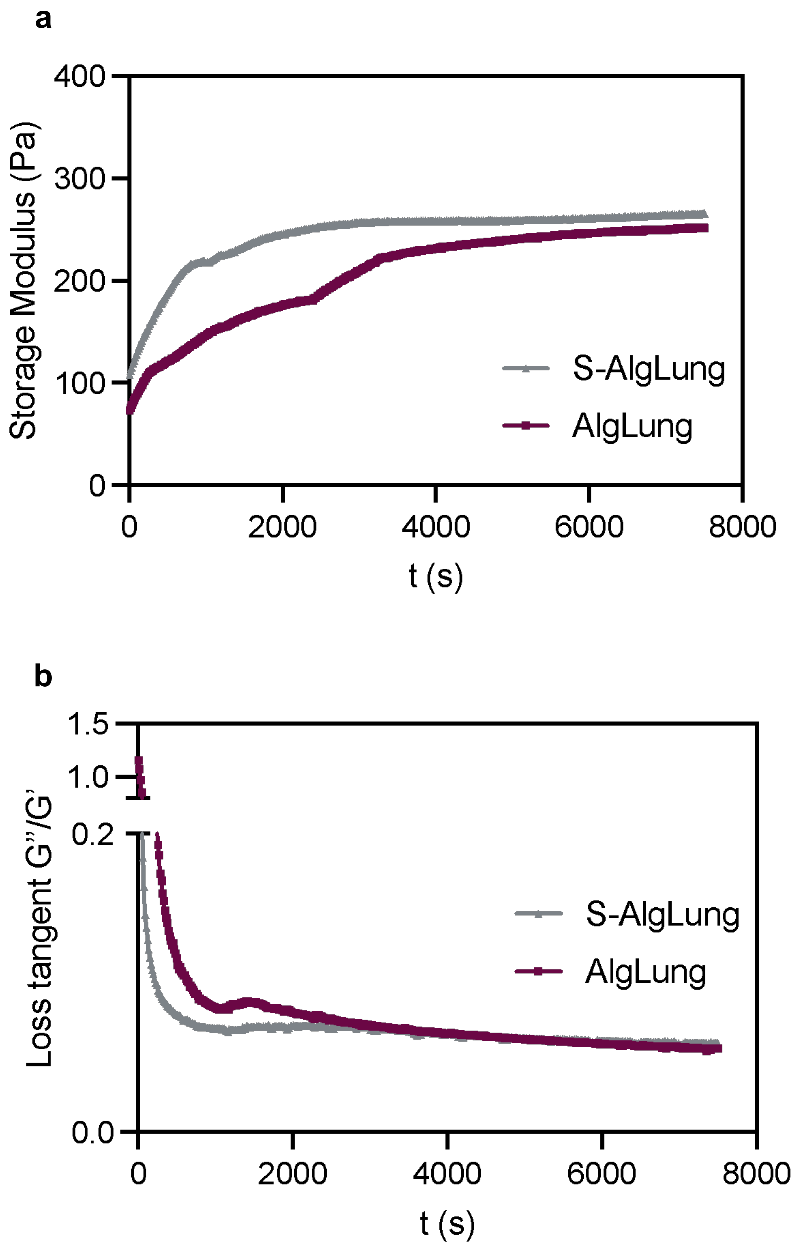

Figure S4. Rheological curves for AlgLung and S-AlgLung hydrogels. a Storage modulus (Pa) and **b** Loss tangent (G''/G') of AlgLung and S-AlgLung hydrogels versus time (s).

Figure S5

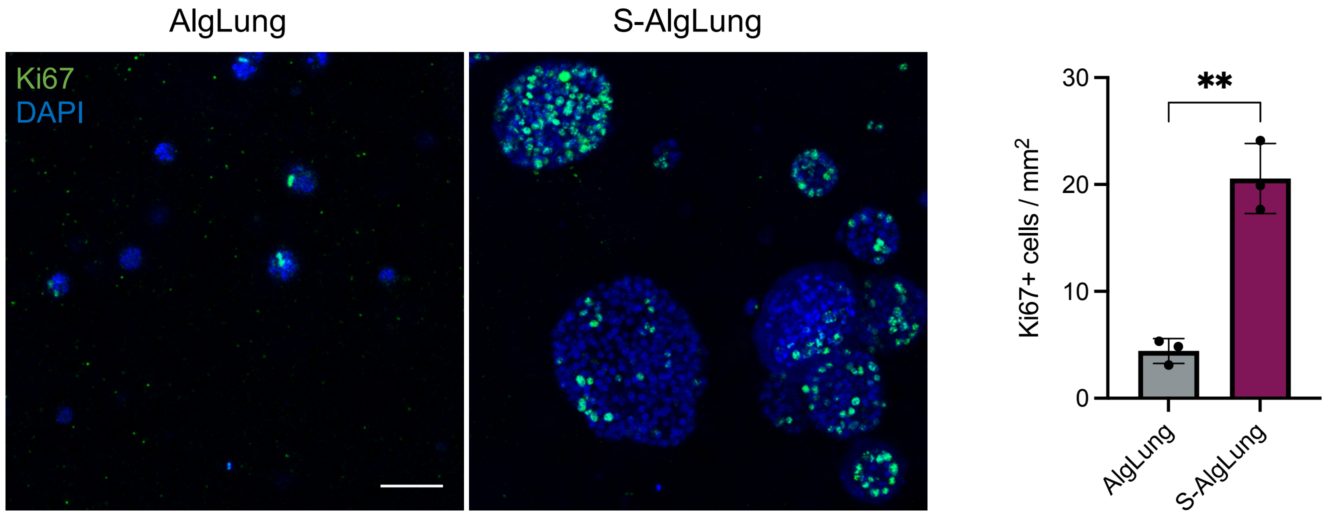

Figure S5. Sulfated ECM induces aberrant proliferation. a Representative immunofluorescence images of Ki67 (green), DAPI (blue) stained A549 cells in AlgLung and S-AlgLung hydrogels (Scale bar: 100 μm). **b** Number of Ki67+ cells were normalized to total cell number per unit area, **p < 0.01.

Figure S6

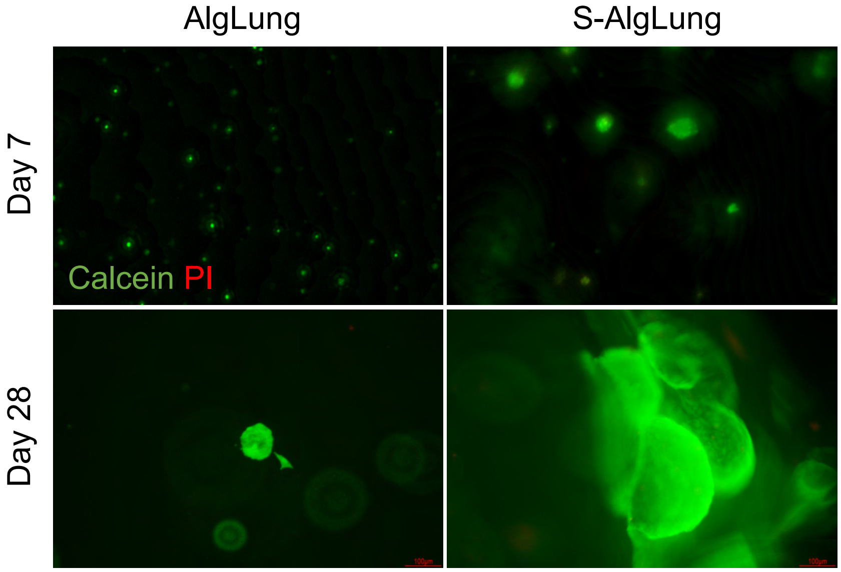

Figure S6. Viability assessment of A549 cells grown in AlgLung and S-AlgLung hydrogels. Calcein AM (green) and propidium iodide (PI) (red) staining of A549 cells grown in AlgLung and S-AlgLung hydrogels at day 7 and day 28 (Scale bar: 100 µm).

Figure S7

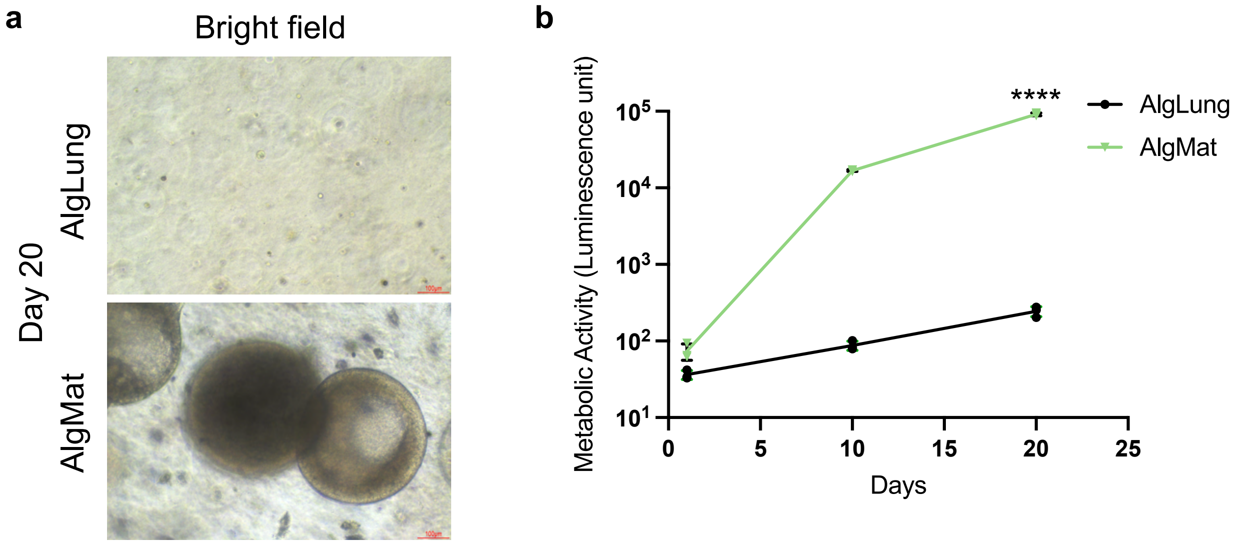

Figure S7. Growth of A549 cells in AlgMat hydrogels. a Representative bright field images of A549 cells grown in AlgLung and AlgMat (Alginate-Matrigel) hydrogels at day 20 (Scale bar: 100 μm). **b** Cellular growth analysis by CellTiter-Glo 3D assay in AlgLung and AlgMat hydrogels. Data is represented as mean ± S.D and statistical significance is analyzed using an unpaired, two-tailed student's t-test, ****$p < 0.0001$.

Figure S8

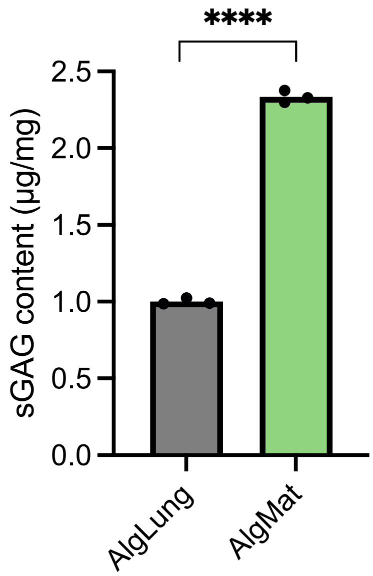

Figure S8. Assessment of sGAG content in AlgLung and AlgMat hydrogels. Data is represented as mean ± S.D and statistical significance is analyzed using an unpaired, two-tailed student's t-test, **** indicates p<0.0001.

Figure S9

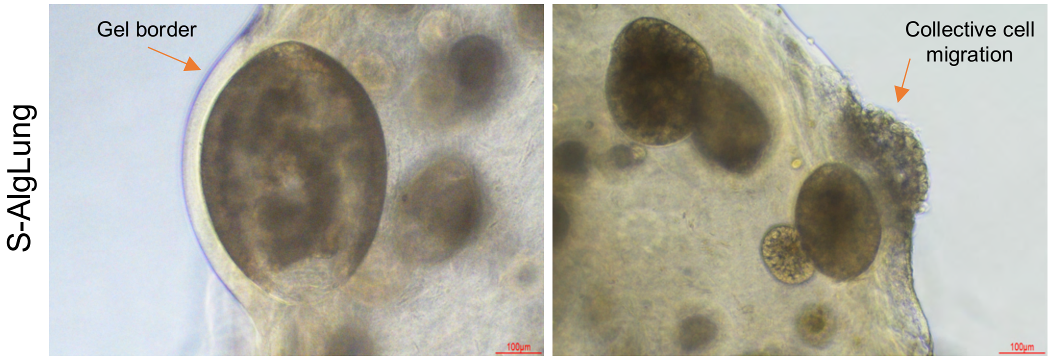

Figure S9. Sulfated hydrogels induce collective cell migration. A549 clusters grown in S-AlgLung hydrogels displayed collective cell migration towards the periphery of the hydrogels, shown via brightfield microscopy images (Scale bar: 100 µm).

Figure S10

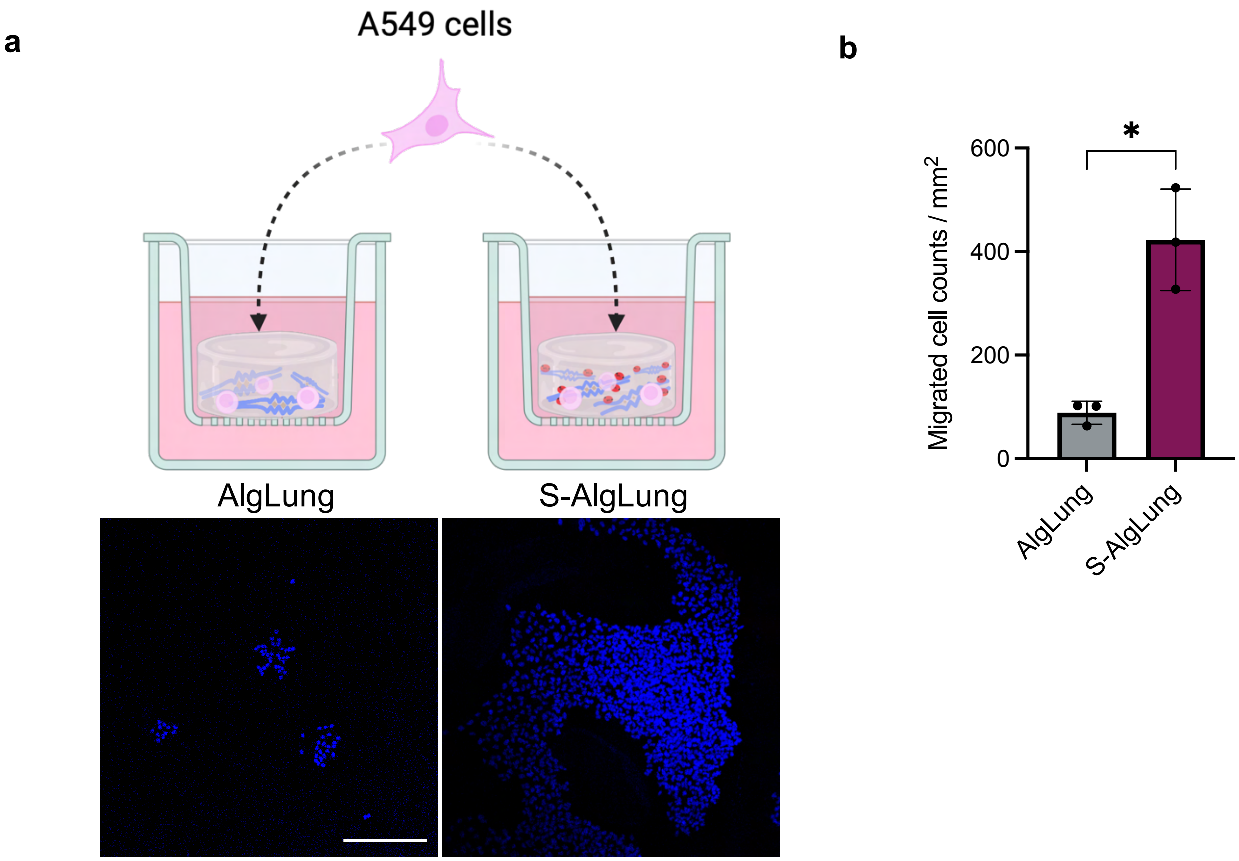

Figure S10. Cells in sulfated ECM have higher migration capacity. a Representative immunofluorescence images of DAPI (blue) stained A549 cells migrated from AlgLung and S-AlgLung hydrogels through transwell pores into the bottom of the plate (Scale bar: 100 μm). **b** Number of migrated cells were counted for 3 different coverslips, *$p < 0.05$**.**

Figure S11

a

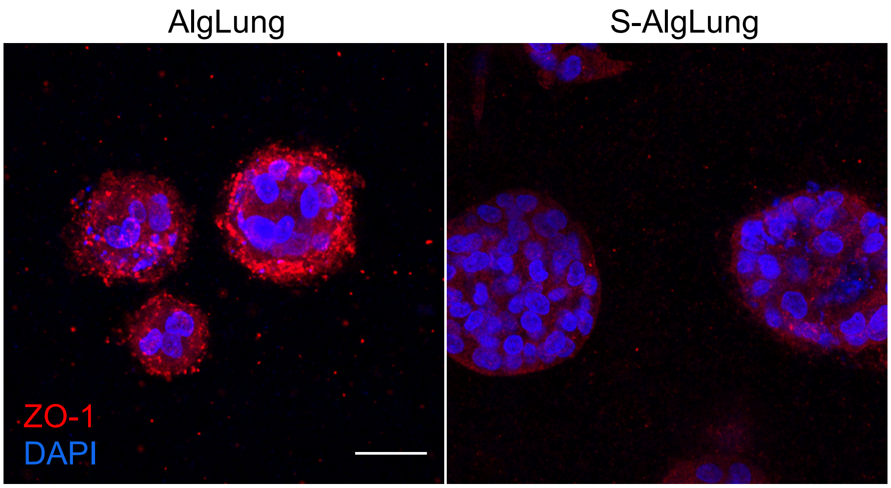

b

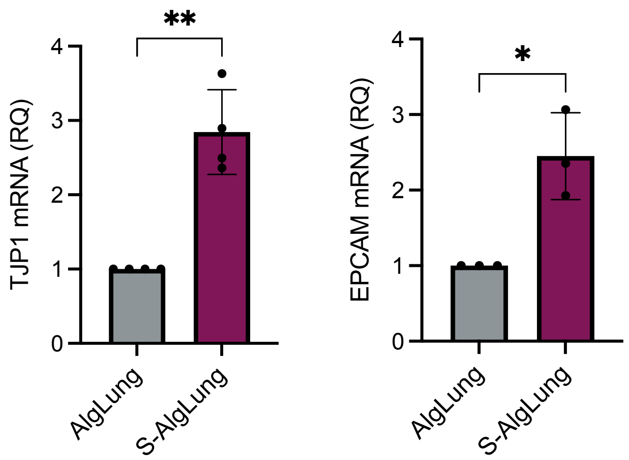

Figure S11. a Representative immunofluorescence images of ZO-1 (red), DAPI (blue) stained A549 cells in AlgLung and S-AlgLung hydrogels (Scale bar: 20 μm). **b** mRNA expression of TJP1 and EPCAM genes in A549 cells cultured in AlgLung and S-AlgLung hydrogels. Relative quantification (RQ) indicates the data is normalized to control group. Data is represented as mean ± S.D and statistical significance is analyzed using an unpaired, two-tailed student's t-test, $*p < 0.05$, $**p < 0.01$.

Figure S12

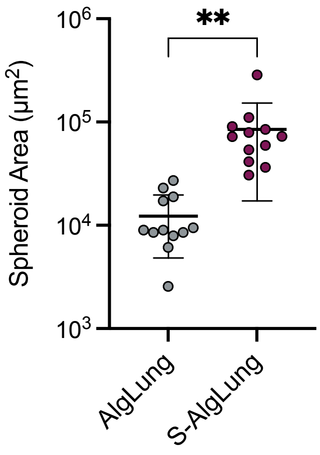

Figure S12. Quantification of spheroid area (µm^2) of cells grown in AlgLung and S-AlgLung hydrogels, **$p < 0.01$

Figure S13

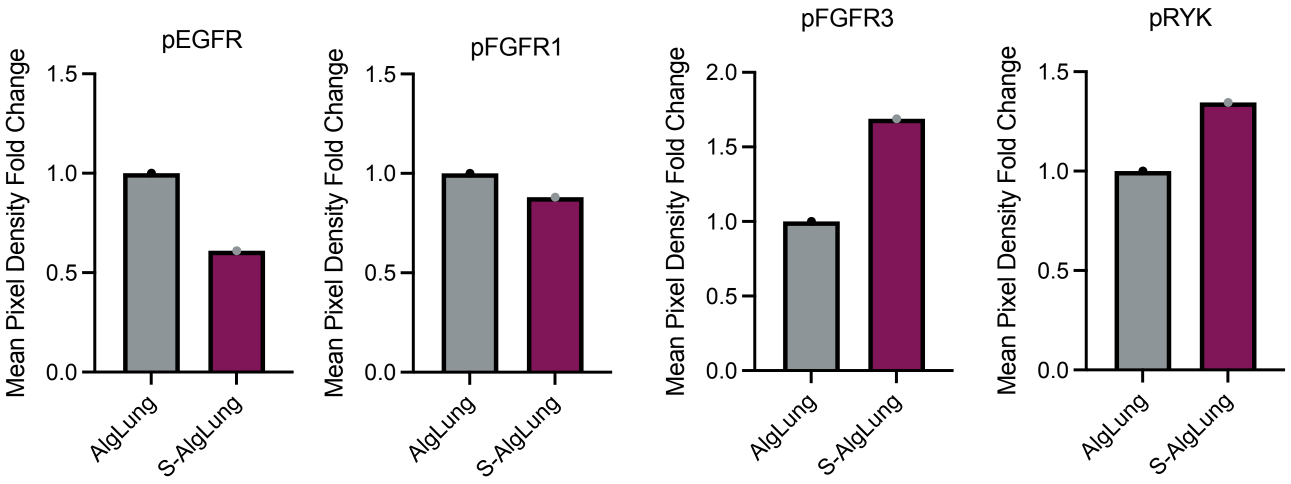

Figure S13. Phosphorylation of EGFR, FGFR1, FGFR3 and RYK. The quantitation of phosphorylated EGFR, FGFR1, FGFR3 and RYK expression from phospho-proteome array (45 min exposure). Pixel density fold change was represented normalized to AlgLung.

Figure S14

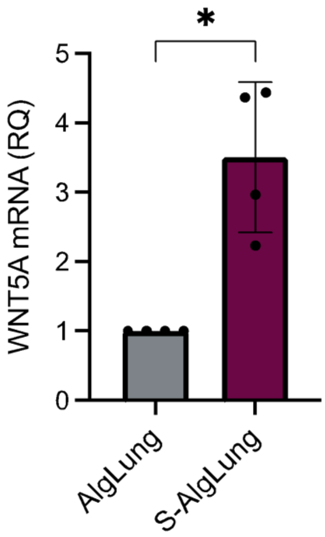

Figure S14. mRNA expression of WNT5A gene in A549 cells cultured in AlgLung and S-AlgLung hydrogels. Relative quantification (RQ) indicates the data is normalized to control group. Data is represented as mean ± S.D and statistical significance is analyzed using an unpaired, two-tailed student's t-test, *p < 0.05.

Figure S15

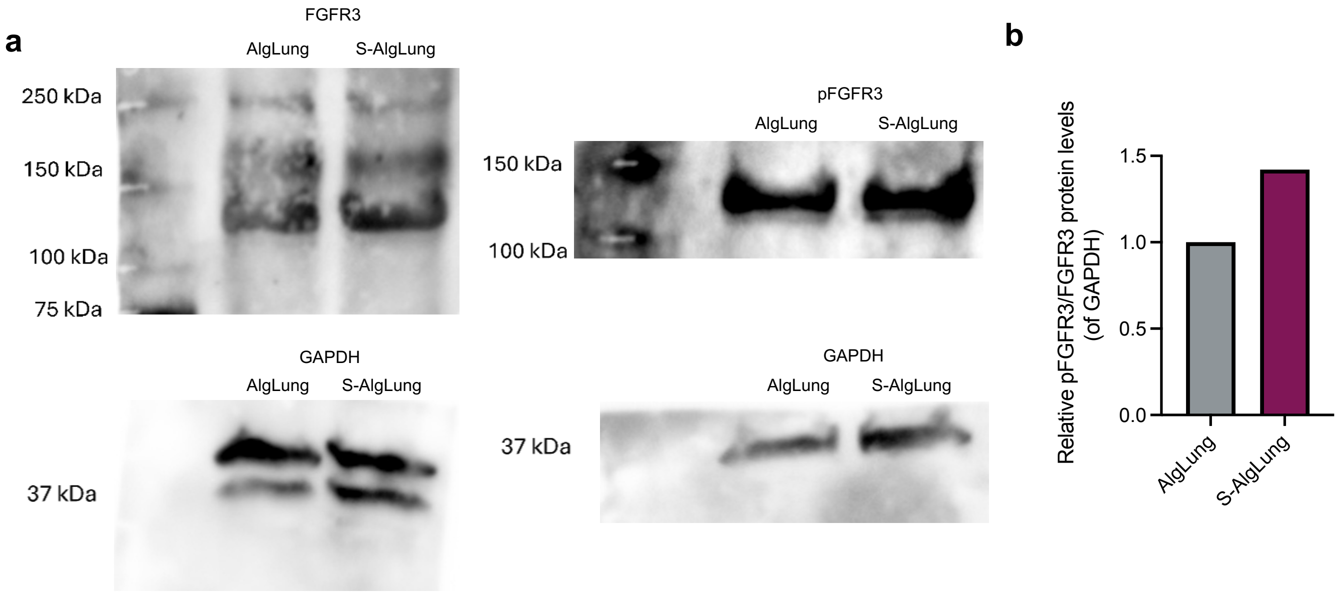

Figure S15. Activation of FGFR3 is validated through the quantification of the total and phosphorylated protein expression by Western Blot Analysis. a Immunoblotting of total FGFR3 and the phosphorylated FGFR3 (pFGFR3) protein expression in A549 cells retrieved from AlgLung and S-AlgLung hydrogels. GAPDH is used as a loading control. **b** Analysis of FGFR3 receptor activation by quantification of the relative pFGFR3/total FGFR3 expression normalized to GAPDH control.

Figure S16

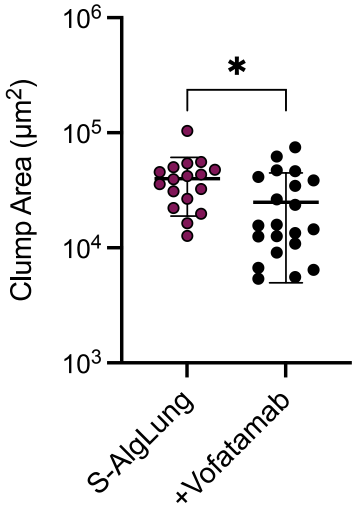

Figure S16. Quantification of clump area (µm^2) of cells grown in S-AlgLung (control) and vofatamab treated hydrogels, **$p < 0.01$

Figure S17

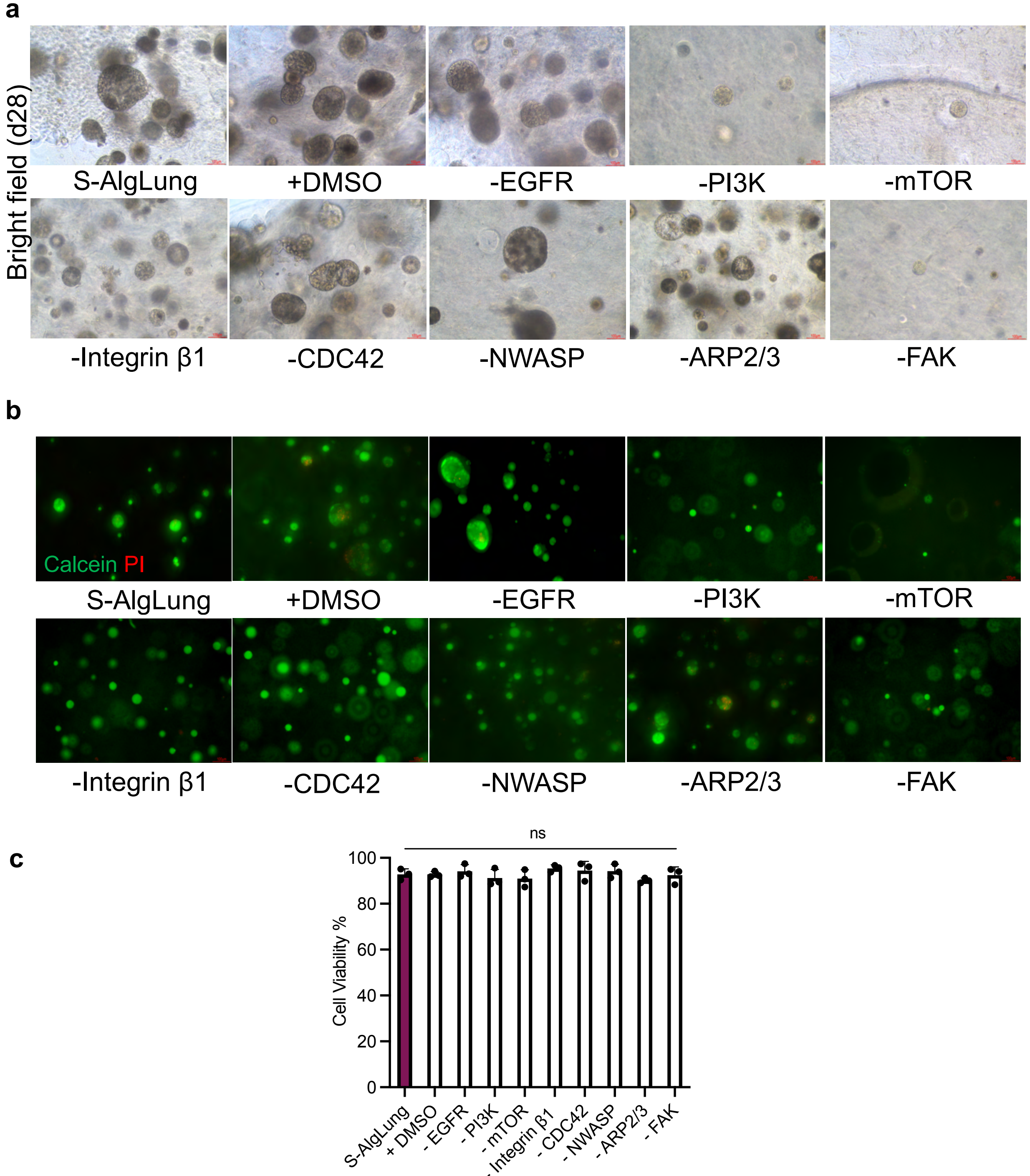

Figure S17. Inhibitor screens. Representative **a** Bright field and **b** immunofluorescence images of calcein AM (green), propidium iodide (red) stained A549 cells in S-AlgLung hydrogels treated with DMSO or inhibitors targeting indicated proteins (Scale bar: 100 μm). **c** Live and dead cells were quantified using ImageJ, ns not significant.

Figure S18

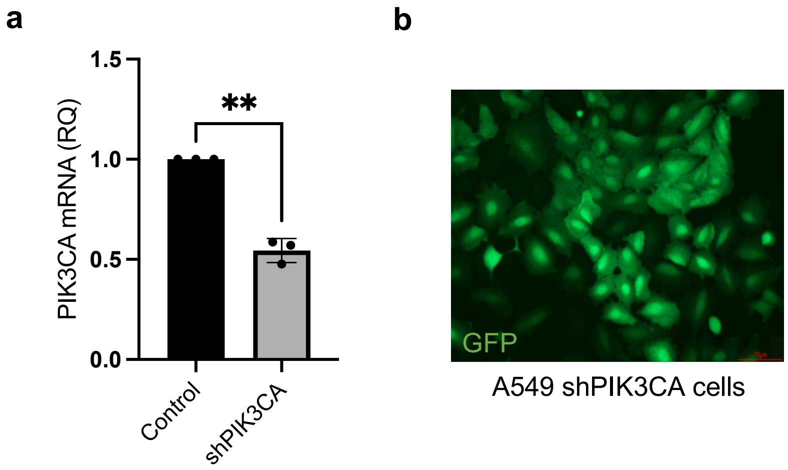

Figure S18. Validation of PI3K knockdown. a mRNA expression of PIK3CA in shPIK3CA expressing A549 cells. Data is represented as mean ± S.D and statistical significance is analyzed using an unpaired, two-tailed student's t-test, **p < 0.01. **b** Representative images of shPIK3CA expressing A549 cells tagged with GFP (Scale bar: 70 μm).

Figure S19

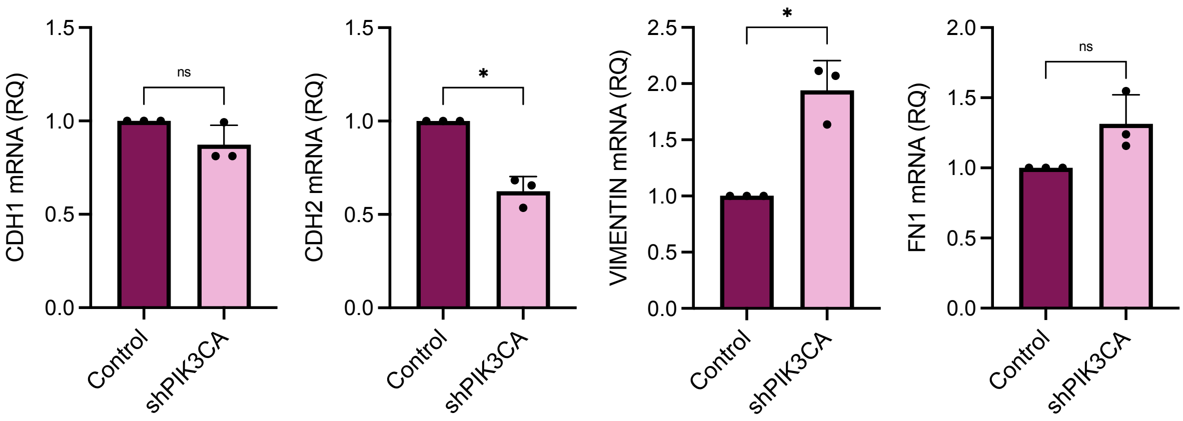

Figure S19. Gene expression of EMT markers in knockdown A549 cells. mRNA expression of E-cadherin, N-cadherin, vimentin and fibronectin in shPIK3CA-expressing A549 cells grown in S-AlgLung hydrogels. Relative quantification (RQ) was used with normalization to control group. Data is represented as mean ± S.D and statistical significance is analyzed using an unpaired, two-tailed student's t-test, ns not significant, *p < 0.05.

Figure S20

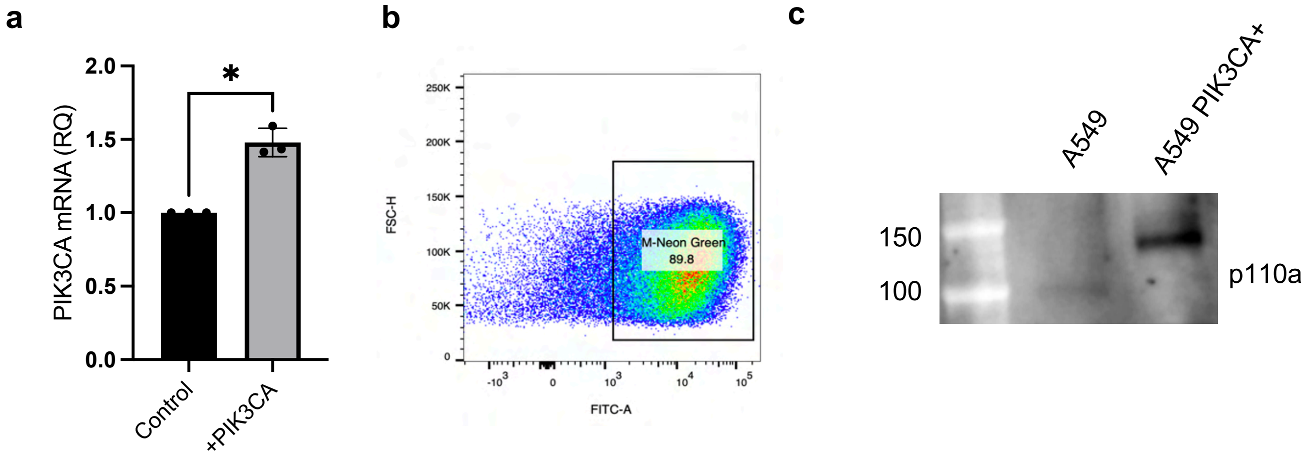

Figure S20. Validation of PIK3CA-overexpressing A549 cell line. a PIK3CA mRNA expression of PIK3CA-overexpressing A549 cells. Data is represented as mean ± S.D and statistical significance is analyzed using an unpaired, two-tailed student's t-test, *p<0.05. **b** FACS plot demonstrating sorting for M-Neon Green-tagged PIK3CA-overexpressing A549 cells. **c** Validation of PIK3CA overexpression through assessment of p110a protein expression in A549 cells by western blotting.

Figure S21

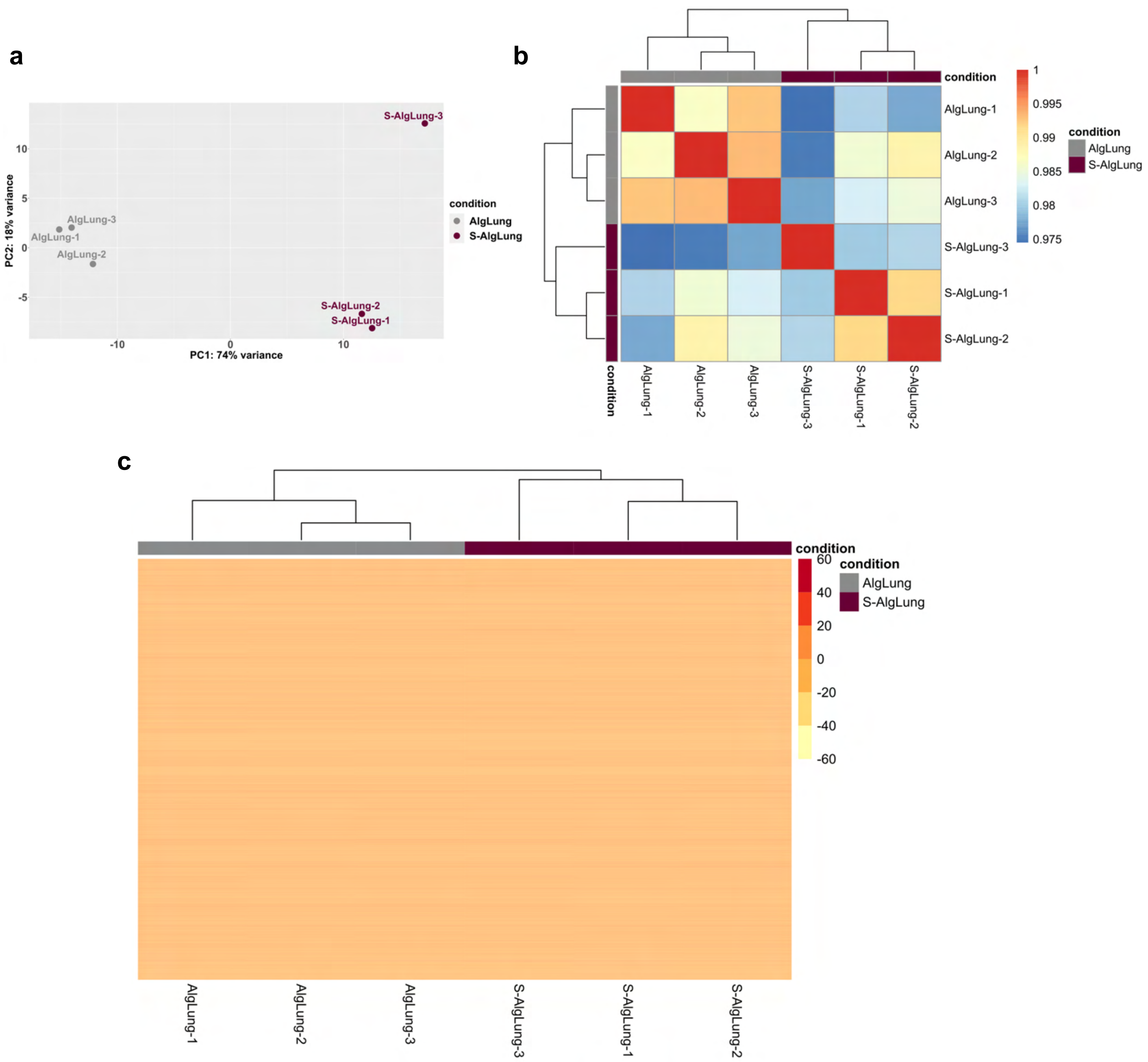

Figure S21. a Principal component analysis (PCA) showed a clear separation of AlgLung from S-AlgLung hydrogels. **b** The correlation heatmap shows the Pearson correlation between samples, where samples of the same condition exhibit higher correlation values. **c** Heatmap shows DESeq2's median of ratios normalized counts for all genes (16,901).

Figure S22

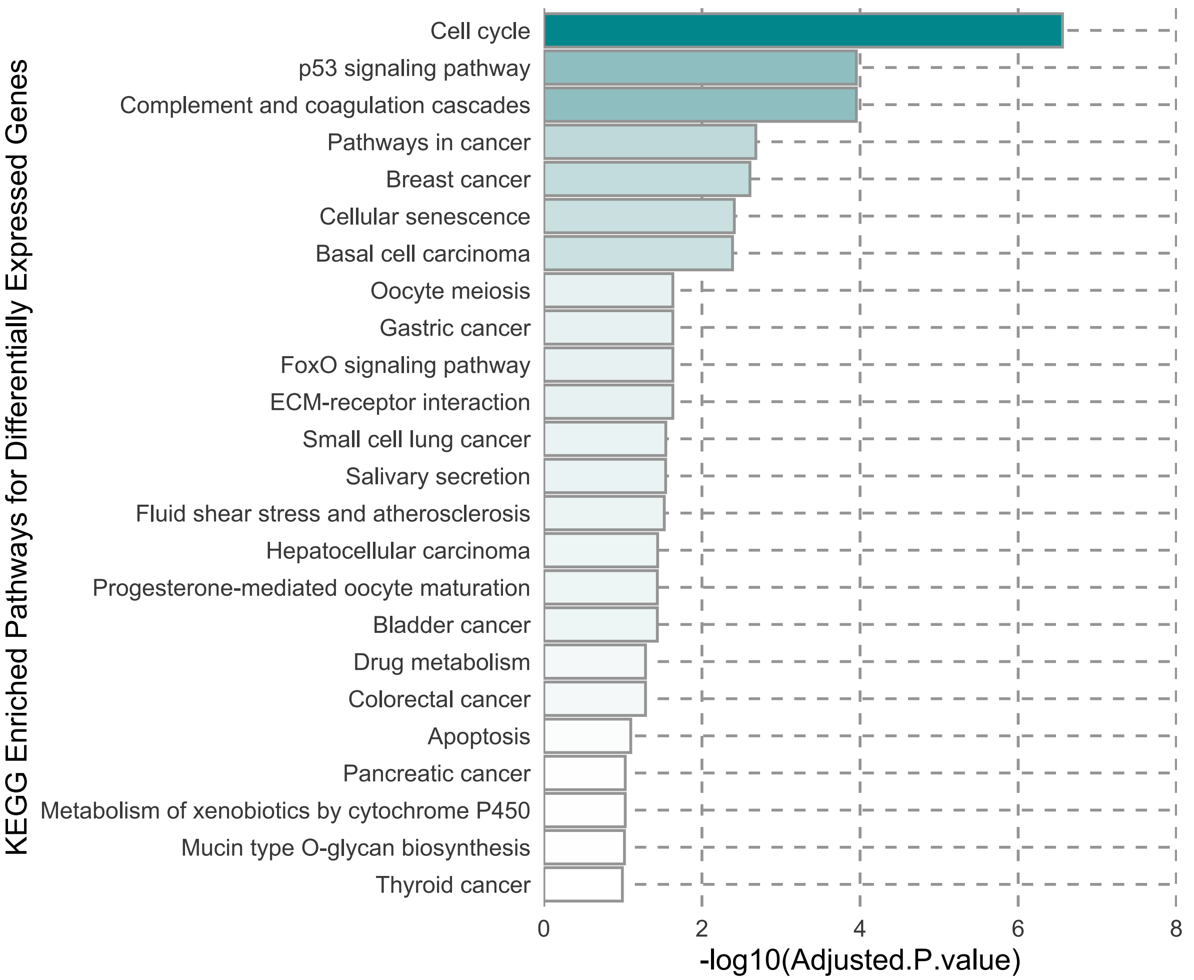

Figure S22. Bar plots showing the functional enrichment of KEGG pathways in up-regulated and down-regulated genes in S-AlgLung hydrogels.

Figure S23

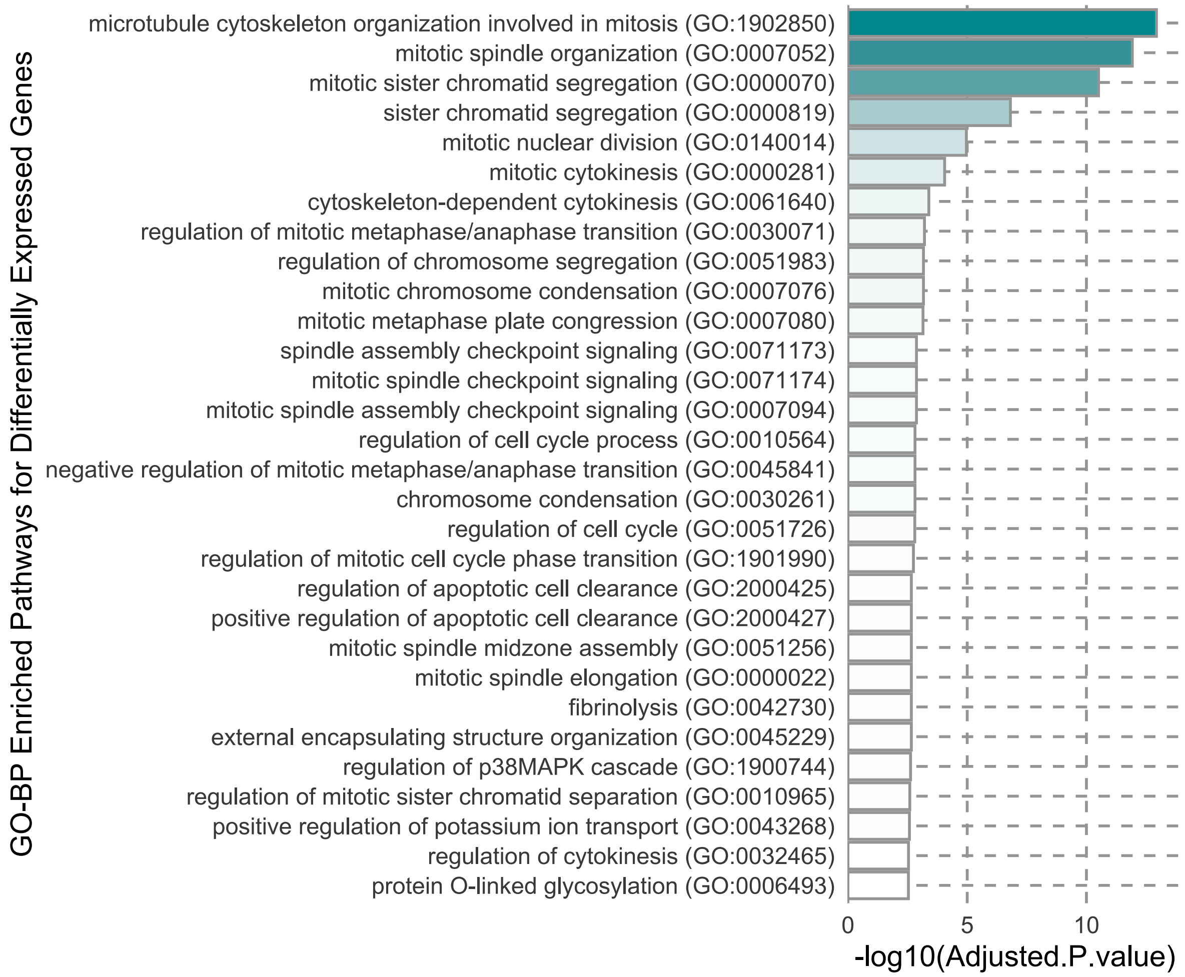

Figure S23. Bar plots showing the functional enrichment of GO biological processes in up-regulated and down-regulated genes in S-AlgLung hydrogels.

Figure S24

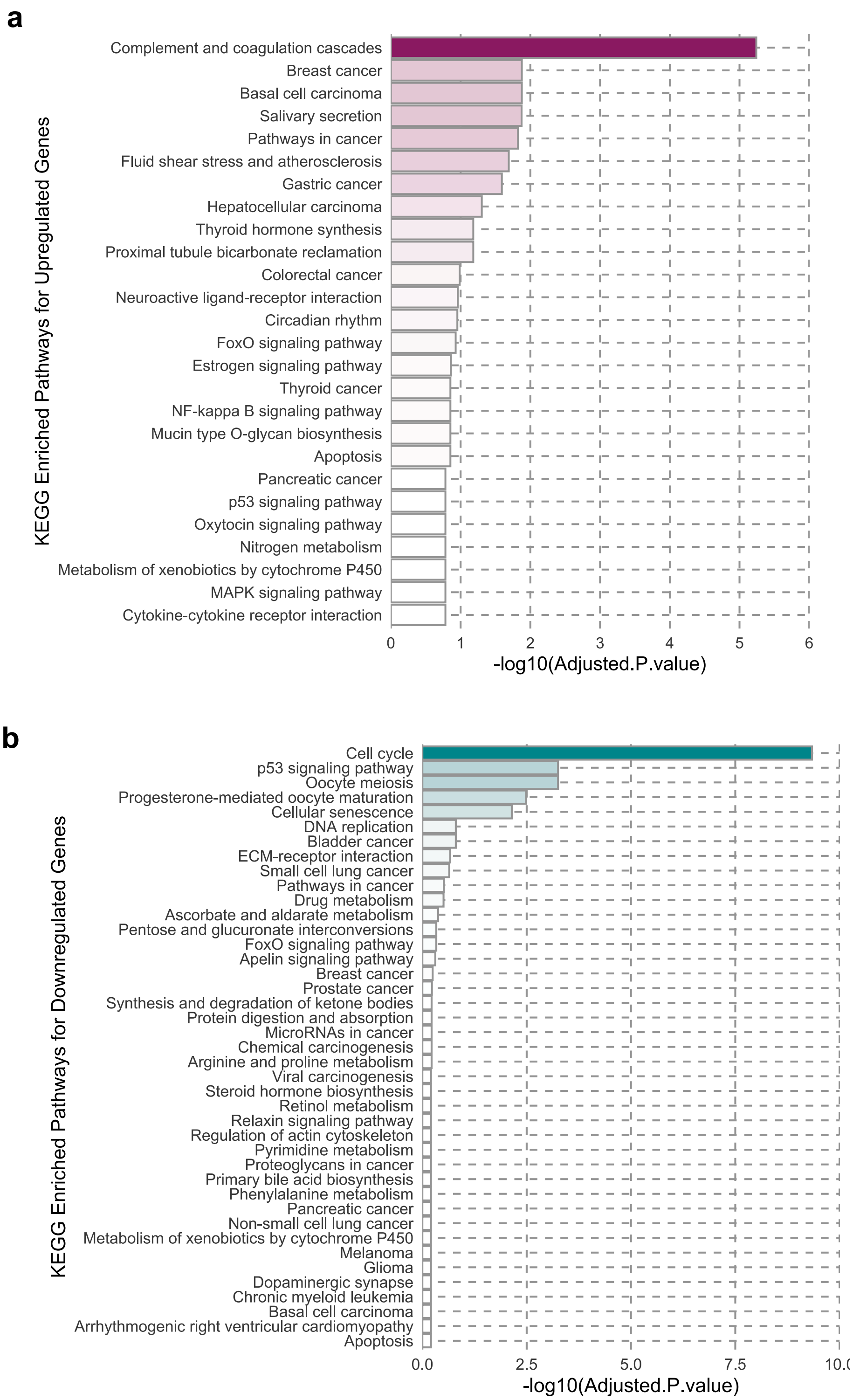

Figure S24. Bar plots showing the functional enrichment of KEGG pathways in **a** up-regulated and, **b** down-regulated genes in S-AlgLung hydrogels.

Figure S25

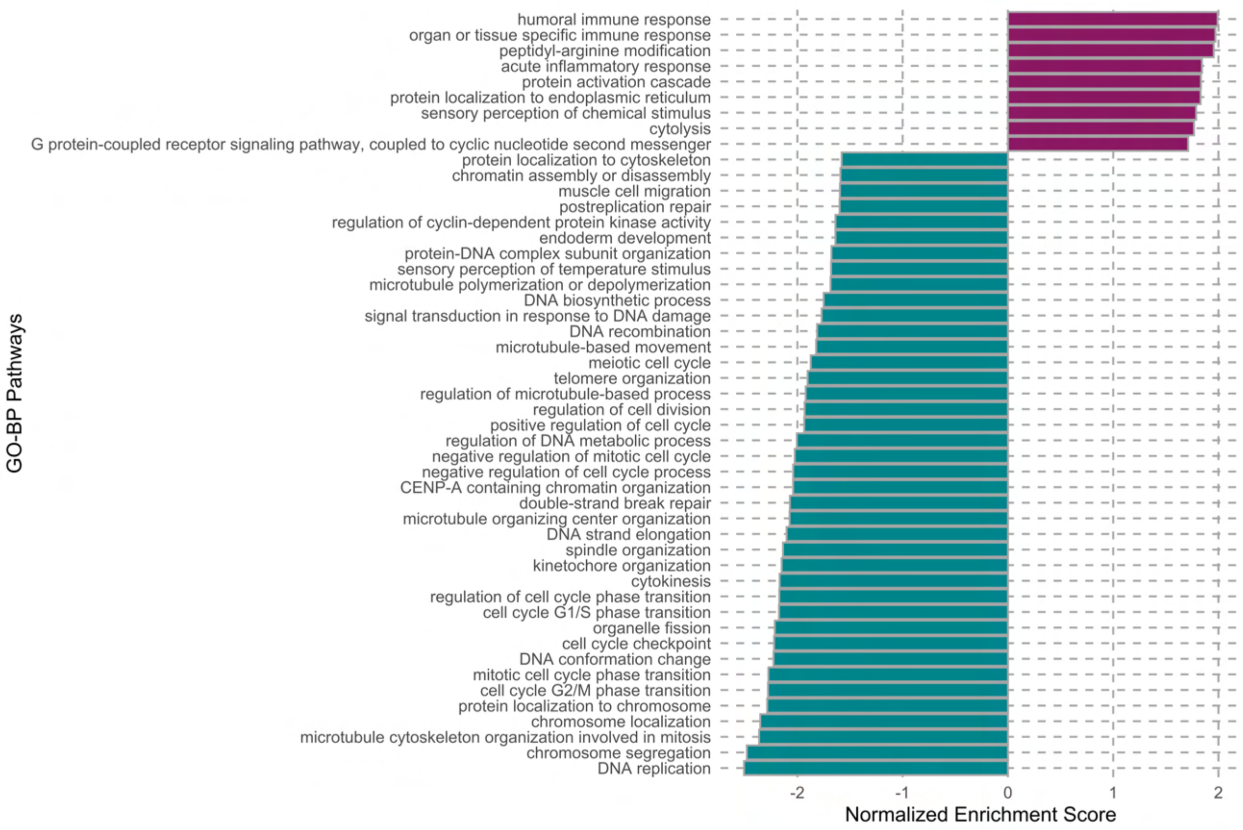

Figure S25. Bar plot showing the gene set enrichment analysis results for GO biological processes (all genes (16,901) and their log2FC were used). X-axis represents the normalized enrichment score where negative values show the down-regulated pathways and positive values show the up-regulated pathways.

Figure S26

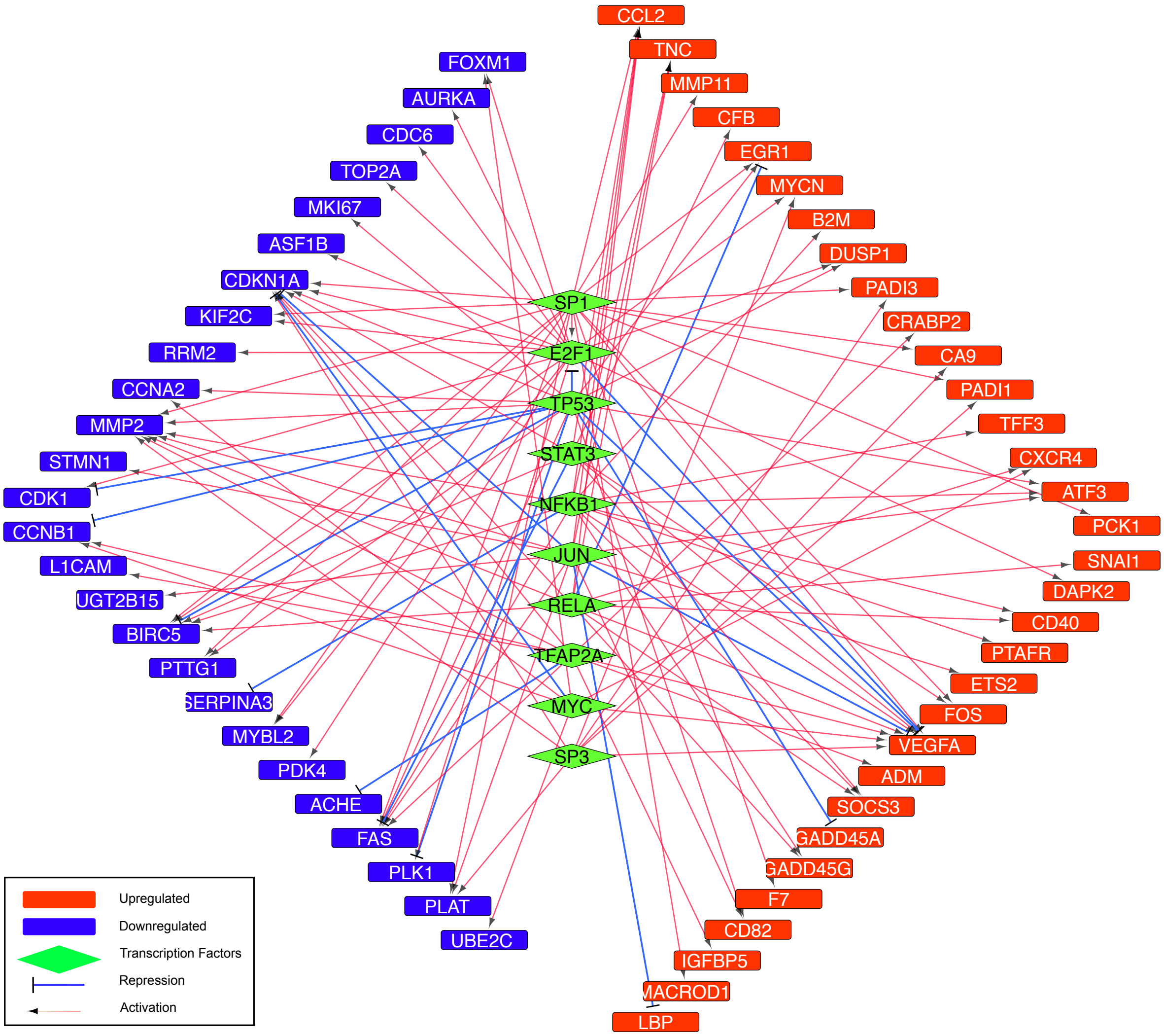

Figure S26. Differentially expressed genes were queried in the TRRUST database in order to reveal their key regulators. To emphasize the most central transcription factors, those with a Q value smaller than 0.01 and a number of overlapping genes higher than 10 are displayed, along with their differentially expressed target genes retrieving the high confidence edge data from the DOROTHEA dataset. MYC is also present as a central transcription factor and it is shown to repress CDKN1A and activate CCNB1, CCNA2, CXCR4, VEGFA, FOXM1, and UBE2C.

Figure S27

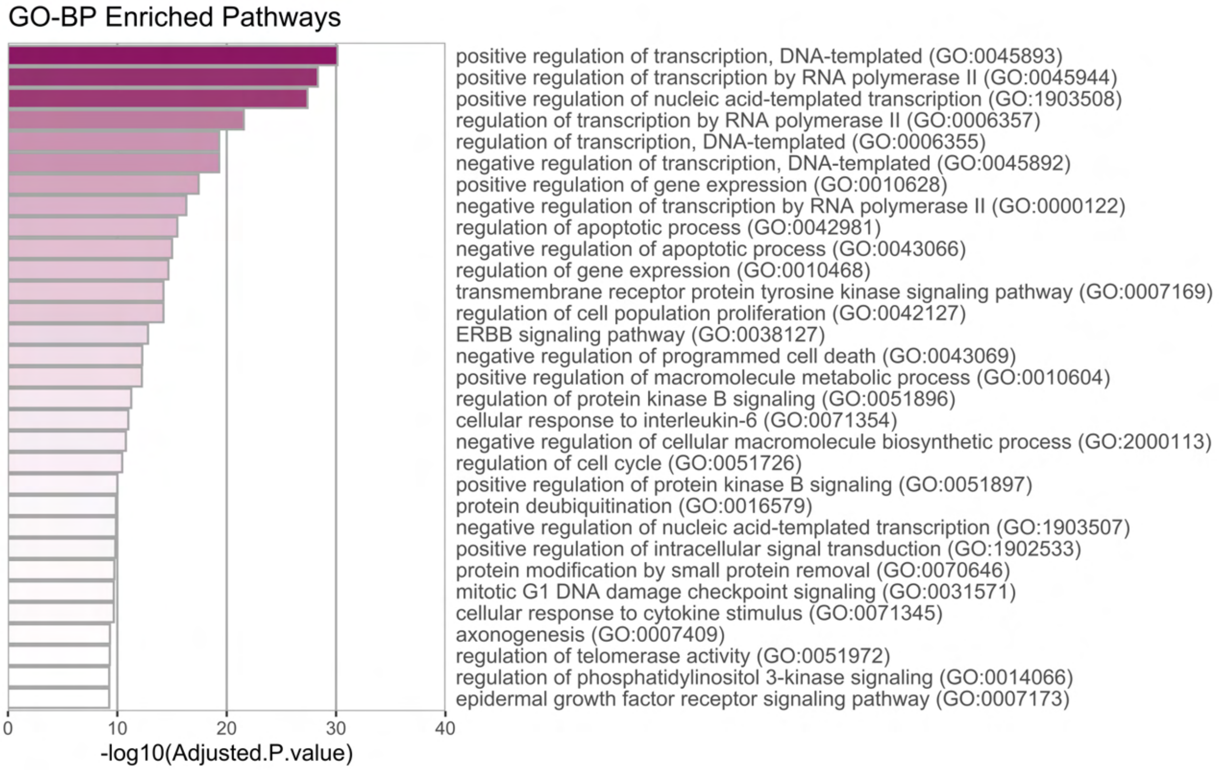

Figure S27. The bar plot shows the functional enrichment of GO biological processes in intermediate proteins revealed from network optimization.

Figure S28

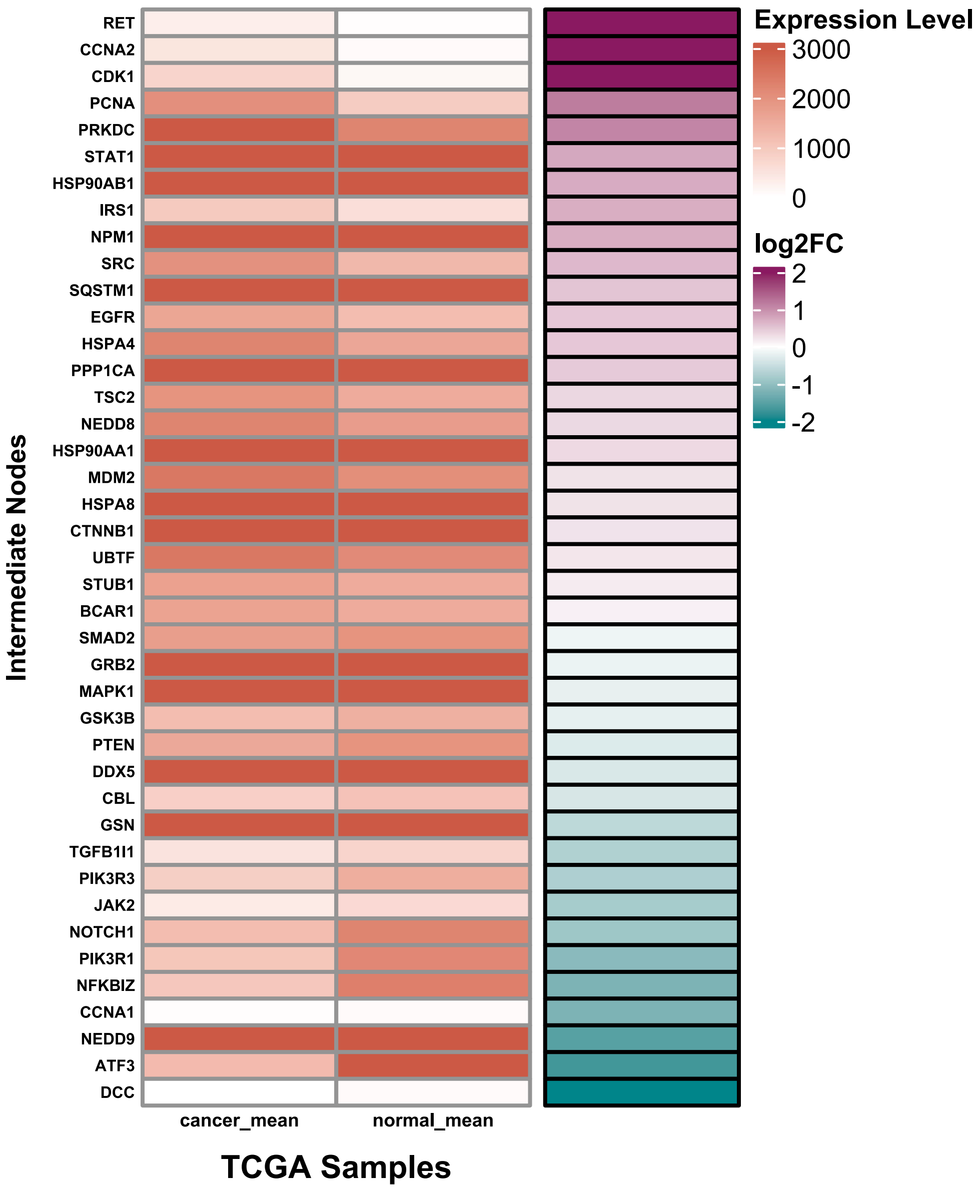

Figure S28. Heatmap shows expression levels of intermediate genes in tumor (510) and normal (59) samples from TCGA (left) and log2 fold change values (right).

Figure S29

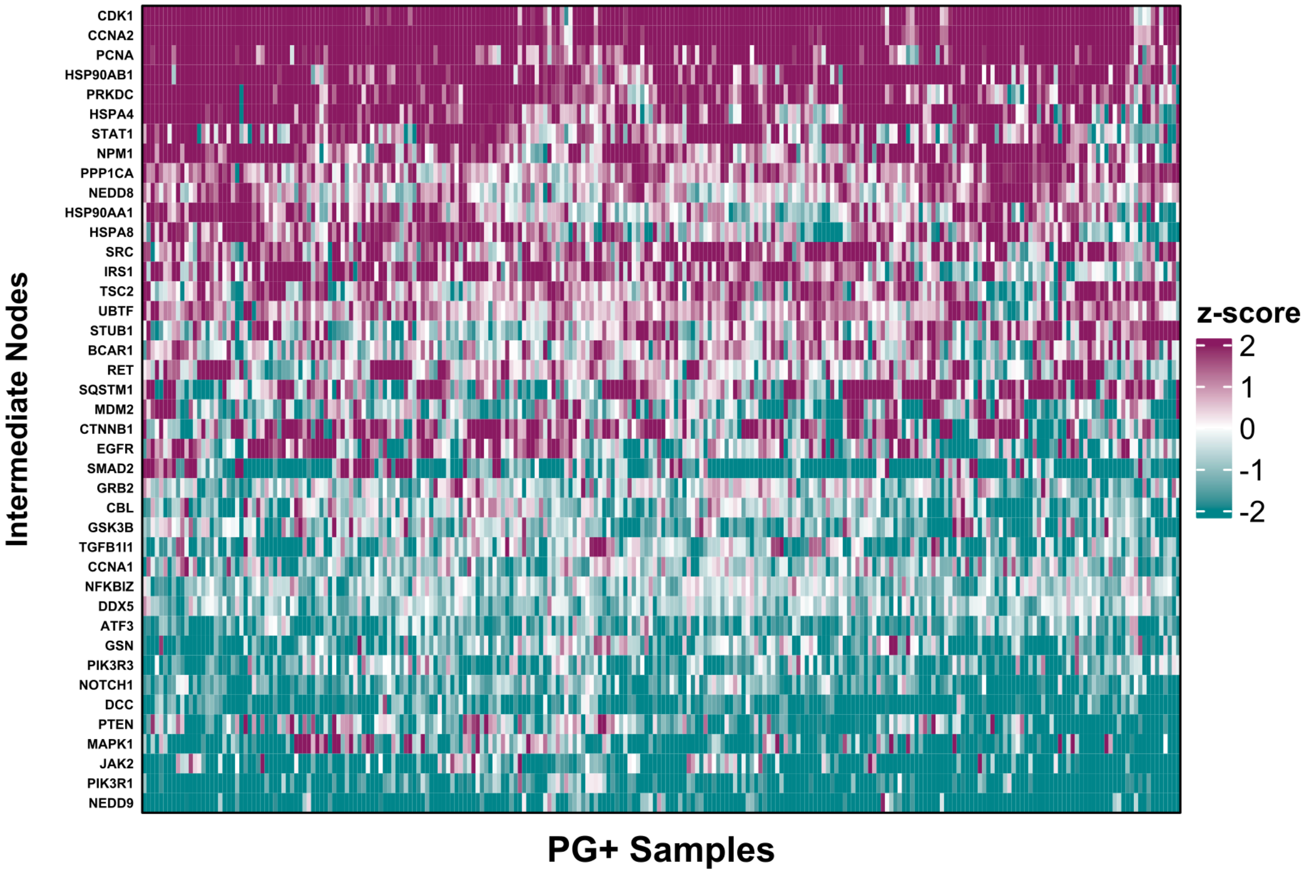

Figure S29. Heatmap shows z-scores of intermediate genes in PG+ TCGA LUAD patient tumor samples. Each column represents a patient.

Figure S30

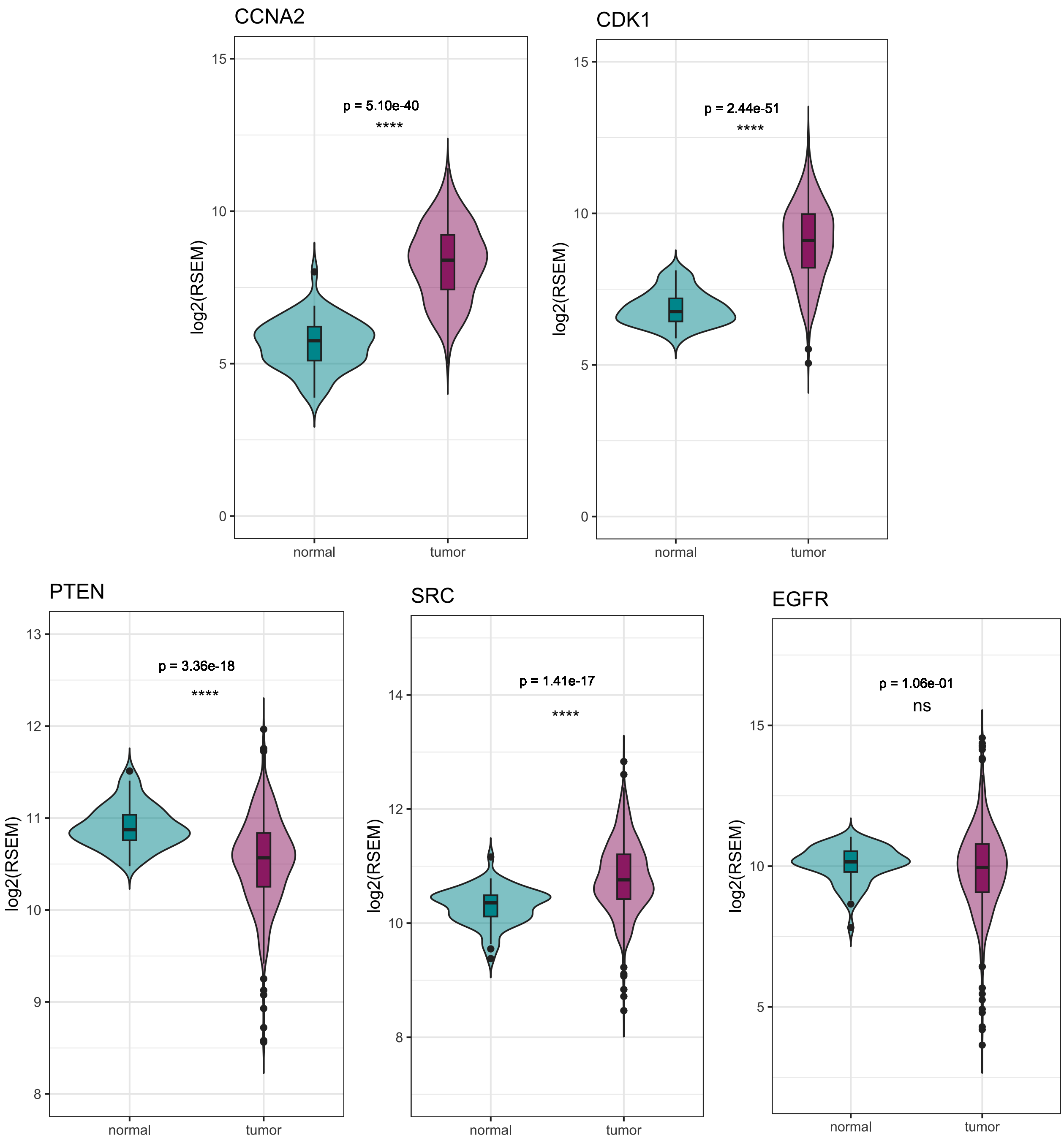

Figure S30. Expression profiles of CCNA2, CDK1, PTEN, SRC and EGFR in TCGA LUAD patient tumor samples compared to normal lung tissue. Statistical analyses were performed using a two-sample t-test. ns indicates non-significant, **** indicates p<0.0001.

Supplementary Tables

Table S1

	Alg			S-Alg		
M_n (kDa)	111,7	107,2	109,5	70,6	67,1	68,9
M_w (kDa)	215,3	211,7	213,5	124,2	124,5	124,4
Polydispersity (M_n/M_w)	1,9	2	2	1,8	1,9	1,9

Table S1. Molecular weight of Alg and S-Alg samples revealed by size exclusion chromatography (SEC-MALS).

Table S2

Proteoglycan Genes	EMT-Related Genes	Invasiveness-Related Genes	CSC-Related Genes
ACAN	CDH1	COL11A1	CD24
ASPN	CDH2	POSTN	CD34
BCAN	FN1	EPYC	CD38
BGN	VIM	ASPN	CD44
CHAD	DSP	COL10A1	CD90
CHADL	OCLN	THBS2	CD133
DCN	MMP1	FAP	KLF4
EPYC	MMP2	LOX	LIN28A
ESM1	MMP3	SFRP4	LIN28B
FMOD	MMP9	INHBA	MYC
HAPLN1	MMP12	MFAP5	NANOG
HAPLN2	SNAI1	GREM1	POU5F1
HAPLN3	SNAI2	COMP	SOX2
HAPLN4	SNAI3	VCAN	FOXP1
HSPG2	TWIST1	COL5A2	NOTCH1
IMPG1	TWIST2	COL5A1	NOTCH2
IMPG2	ZEB1	TIMP3	DLL1
KERA	ZEB2	GAS1	DLL4
LUM	SPARC	TNFAIP6	DDR1
NCAN	TIMP1	ADAM12	DDR2
NYX	SERPINE1	FBN1	DKK1
OGN		SULF1	FZD7
OMD		COL1A1	WNT1
PODN		DCN	SMO
PODNL1			ABCB1
PRELP			ABCB5
PRG2			ALDH1A1
PRG3			ALDH1A3
PRG4			ALDH3A1
SPOCK1			BMI1
SPOCK2			ENG
SPOCK3			KIT
SRGN			AXL
VCAN			FOXA2
VCAN			

Table S2. Proteoglycan, EMT, Invasiveness and CSC-related genes used in bioinformatic analyses.

Table S3

	Age	Gender	Diagnosis
Patient #1	63	Male	Lung Adenocarcinoma
Patient #2	63	Female	Lung Adenocarcinoma
Patient #3	52	Female	Lung Adenocarcinoma
Patient #4	69	Female	Lung Adenocarcinoma
Patient #5	59	Female	Lung Adenocarcinoma

Table S3. Age and gender information of patient donors whose tumor and normal lung parenchyma tissues were used to perform histological stainings and sGAG assay in Figure 1.

Table S4

Key TF	Description	# of overlapped genes	P value	Q value	List of overlapped genes
TP53	tumor protein p53	22	2.12e-11	1.66e-9	BIRC5,FAS,CCNA2,CCNB2,CD82,MKI67,PLK1,ATF3,KLF2,PTTG1,GADD45A,PRC1,VEGFA,CDKN1A,RECQL4,FOXM1,EGR1,E2F1,CCNB1,DUSP1,MMP2,CDK1
E2F1	E2F transcription factor 1	20	2.42e-11	1.66e-9	MYBL2,CCNB1,CDK1,E2F1,FOXM1,RRM2,CDC6,CDKN1A,AURKB,BIRC5,AURKA,PDK4,DUSP1,PLK1,KIF2C,VEGFA,DAPK2,TOP2A,ASF1B,MYCN
E2F3	E2F transcription factor 3	8	7.61e-11	3.48e-9	CCNB1,PLK1,CDC6,CDKN1A,AURKA,CDK1,CCNA2,VEGFA
NFKB1	nuclear factor of kappa light polypeptide gene enhancer in B-cells 1	26	6.7e-9	2.29e-7	PTGDS,EGR1,PDK4,CXCR4,SERPINA3,TNC,SNAI1,PSMB9,TFF3,ATF3,SOCS3,CDKN1A,CCNB1,MMP2,VEGFA,CD40,E2F1,PTAFR,MUC5B,GADD45G,B2M,POU2F2,BIRC5,CCL2,FAS,HES1
YBX1	Y box binding protein 1	9	1.36e-8	3.73e-7	TOP2A,CCNB1,MMP2,E2F1,CDC20,FAS,MYCN,CXCR4,ERBB3
ATF2	activating transcription factor 2	9	2.57e-8	5.86e-7	PCK1,CDKN1A,ATF3,JUN,PLAT,FAS,ITGB8,MMP2,DUSP1
TFAP2A	transcription factor AP-2 alpha (activating enhancer binding protein 2 alpha)	12	6.01e-8	1.18e-6	CDKN1A,VEGFA,MMP2,FAS,CRABP2,L1CAM,CD82,CCNB1,ACHE,ADM,MCAM,IGFBP5
SP1	Sp1 transcription factor	31	1.23e-7	2.11e-6	A,UGT2B15,MYCN,GIPR,CCL2,MMP2,MYBL2,TNC,EGR1,FOXM1,FBN1,MACROD1,CA9,RECQL4,VEGFA,PTTG1,C4B,KIF2C,BIRC5,E2F1,CDKN1A,FAS,MMP11,PLAT,PADI1,CCNA2,TK1,PADI3,F7,FOS,PC
JUN	jun proto-oncogene	16	2.83e-7	4.31e-6	PLAT,CDKN1A,FAS,CCL2,MMP2,ITGB8,CD82,ATF3,ETS2,STMN1,TNC,NEFL,UGT2B15,JUN,VEGFA,LBP
ATM	ataxia telangiectasia mutated	7	3.72e-7	5.1e-6	FOXM1,FAS,PBK,CDKN1A,DUSP1,GADD45A,VEGFA
RELA	v-rel reticuloendotheliosis viral oncogene homolog A (avian)	23	4.11e-7	5.12e-6	TFF3,EGR1,VEGFA,FAS,E2F1,CD40,MMP2,PDK4,BIRC5,PSMB9,HES1,CCL2,POU2F2,TNC,CDKN1A,CXCR4,MUC5B,GADD45G,PTAFR,SNAI1,SERPINA3,CCNB1,SOCS3
E2F4	E2F transcription factor 4, p107/p130-binding	7	5.25e-7	5.99e-6	PCLAF,PLK1,BIRC5,TTK,CCNB1,MCM10,AURKB
KLF4	Kruppel-like factor 4 (gut)	7	2.86e-5	2.83e-4	BIRC5,VEGFA,MMP2,ATF3,LAMA3,CCNB1,CDKN1A
FOXM1	forkhead box M1	5	2.89e-5	2.83e-4	CCNB1,FGB,CDC6,VEGFA,KRT15
HDAC1	histone deacetylase 1	9	3.12e-5	2.85e-4	E2F1,RECQL4,EGR1,CD82,TXNIP,SFRP1,BIRC5,FOS,CDKN1A
BRCA1	breast cancer 1, early onset	8	4.08e-5	3.49e-4	CDKN1A,FST,GADD45A,EGR1,ASPM,CCNB1,VEGFA,FOS
SP3	Sp3 transcription factor	11	5.2e-5	4.19e-4	CA9,GIPR,MYCN,CDKN1A,SOCS3,PADI3,PADI1,PLAT,MMP2,VEGFA,BIRC5
RB1	retinoblastoma 1	6	5.97e-5	4.47e-4	CDKN1A,VEGFA,FOS,E2F1,BIRC5,CDK1
MYCN	-myc myelocytomatosis viral related oncogene, neuroblastoma derived (aviar	7	6.32e-5	4.47e-4	MCM10,CDKN1A,MYBL2,MCM2,BIRC5,MCM4,HLA-B
KLF6	Kruppel-like factor 6	5	6.85e-5	4.47e-4	ATF3,CDKN1A,PTTG1,DAPK2,TXNIP
VHL	von Hippel-Lindau tumor suppressor, E3 ubiquitin protein ligase	5	6.85e-5	4.47e-4	COL4A2,CA9,CXCR4,KLF10,VEGFA
MYC	v-myc myelocytomatosis viral oncogene homolog (avian)	10	9.05e-5	5.64e-4	VEGFA,CCNB1,HLA-B,ATF3,CXCR4,CCNA2,SFRP1,FOXM1,UBE2C,CDKN1A
STAT3	ignal transducer and activator of transcription 3 (acute-phase response facto	12	9.61e-5	5.73e-4	FOS,BIRC5,CFB,MUC5B,MMP2,FGG,VEGFA,FAS,CDKN1A,SOCS3,S1PR1,CCL2
PARP1	poly (ADP-ribose) polymerase 1	5	2.14e-4	0.00112	FBN1,SNAI1,CDKN1A,JUN,E2F1
TCF4	transcription factor 4	5	2.14e-4	0.00112	JUN,BIRC5,WNT4,FST,VEGFA
AR	androgen receptor	9	2.6e-4	0.00123	ERBB3,CDC6,HMMR,CDKN1A,JUN,PIP,UGT2B15,KISS1R,VEGFA
EP300	E1A binding protein p300	7	2.61e-4	0.00123	VEGFA,CA9,BIRC5,NR0B2,CCNB2,LAMA3,CDKN1A
SMAD3	SMAD family member 3	5	6.12e-4	0.0025	VEGFA,TNC,JUN,FST,CDKN1A
ETS2	v-ets erythroblastosis virus E26 oncogene homolog 2 (avian)	5	7.12e-4	0.00279	EGR1,CDKN1A,CDK1,MMP2,TNC
IRF1	interferon regulatory factor 1	6	9.97e-4	0.00334	CDKN1A,CDK1,PSMB9,E2F1,CD40,CCNB1
CREB1	cAMP responsive element binding protein 1	8	1	0.00334	PLAT,FOS,MMP2,CXCR4,STMN1,MSMB,JUN,SNAI1
ATF4	activating transcription factor 4 (tax-responsive enhancer element B67)	5	0.00108	0.00354	F7,ATF3,CA9,VEGFA,CCL2
ESR1	estrogen receptor 1	7	0.00167	0.00519	VEGFA,CDKN1A,E2F1,CCNA2,UGT2B15,JUN,FOS
SPI1	spleen focus forming virus (SFFV) proviral integration oncogene spi1	6	0.00276	0.00766	CD40,DAPK2,PRTN3,CCL2,ELANE,MACROD1

Table S4. List of transcription factors with a Q value smaller than or equal to 0.01 and the number of overlapped genes higher than or equal to 5 retrieved from the TRRUST database using the differentially expressed genes and provided as target nodes to Pathlinker for network construction.

Table S5

Protein	Antibody
EGFR	Biolegend (352901)
Integrin β1/ITGB1	SantaCruz (sc-13590L)
E-Cadherin	Cell Signaling Technologies (3195)
Vimentin	Cell Signaling Technologies (5741)
N-Cadherin	Cell Signaling Technologies (13116)
Fibronectin	Abcam (ab2413)
β-Catenin	Abcam (ab6302)
SOX2	Biolegend (630801)
MUC5B	Sigma (HPA008246)
P63	Cell Signaling Technologies (13109)
TTF-1	Cell Signaling Technologies (12373)
ZO-1	Cell Signaling Technologies (13663)
Ki67	Cell Signaling Technologies (9129)
FGFR3	Abclonal (A0404)
pFGFR3-Y724	Abclonal (AP1274)

Table S5. List of antibodies used in immunofluorescence.

Table S6

Target	Inhibitor	Concentration
EGFR	Erlotinib	5 uM
FGFR	PD173074	250 nM
FGFR3	Vofatamab	1 ug/ml
TGFBR	A83-01	250 nM
Integrin β1 (P5D2)	SC13590	1 ug/ml
PI3K	LY294002	20 uM
CDC42	ML141	10 uM
ARP2/3	CK-666	100 uM
NWASP	Wiskostatin	10 uM
FAK	PF-573228	5 uM
mTOR	Rapamycin	250 nM

Table S6. List of inhibitors and their final concentrations used in inhibition assays.

Table S7.

PI3KCA_human_forward	CCATATCTCCAAAGTAGAAC	VIMENTIN_human_forward	ACCAGCTAACCAACGACAAAG
PI3KCA_human_reverse	TAGAATGACGACTAGCAGTA	VIMENTIN_human_reverse	AAAGATTGCAGGGTGTTTTCG
ZEB1_human_forward	ACCCTTGAAAGTGATCCAGC	FN1_human_forward	CAGAGGCATAAGGTTCGGG
ZEB1_human_reverse	CATTCCATTTTCTGTCTTCCGC	FN1_human_reverse	TTCAGACATTCGTTCCCACTC
ZEB2_human_forward	ACTCCTGTCTGTCTCGCAAA	SOX2_human_forward	CTTTTATGAGAGAGATCCTG
ZEB2_human_reverse	GCTCGATAAGGTGGTGCTTG	SOX2_human_reverse	ACCGTACCACTAGAACTTT
SNAIL_human_forward	GGAAGCCTAACTACAGCGAG	OCT3/4_human_forward	CACTAAGGAAGGAATTGG
SNAIL_human_reverse	CAGAGTCCCAGATGAGCATTG	OCT3/4_human_reverse	GTGTGTCTATCTACTGTGTCC
SLUG_human_forward	AGCATTTCAACGCCTCCA	KLF4_human_forward	TCTGTGACTGGATCTTCTAT
SLUG_human_reverse	GGATCTCTGGTTGTGGTATGAC	KLF4_human_reverse	CTTCCTCTTCTTCTAACATC
TWIST1_human_forward	GCATTCTCAAGAGGTCGTGC	CD44_human_forward	CTCACTCAAGCTCTTTAACT
TWIST1_human_reverse	TGACTATGGTTTTGCAGGCC	CD44_human_reverse	GAATATCTAGAAGGAGTGGA
TWIST2_human_forward	AGAGCGACGAGATGGACAAT	CD133_human_forward	ACCTACAGCATATTCTTCAC
TWIST2_human_reverse	ACAGACTCGAATGCATCCCA	CD133_human_reverse	CTGTTAAACTGTACACCGTA
CDH1_human_forward	CTGCCAATCCCGATGAAATTG	MUC5AC_human_forward	ACCAGCATCTTCATCAACCT
CDH1_human_reverse	TCCTTCATAGTCAAACACGAGC	MUC5AC_human_reverse	AAGTCATTAACGGCGATGTC
CDH2_human_forward	CAGAATCAGTGGCGGAGATC	MUC5B_human_forward	CTACATCAAGGTCAGCATCCG
CDH2_human_reverse	CAGCAACAGTAAGGACAAACATC	MUC5B_human_reverse	ATAGAACTCGTTGAAGGCCG
WNT5A_human_forward	CCCCCTTATAAATGCAACTGTTC	GAPDH_human_forward	CTGACTTCAACAGCGACACC
WNT5A_human_reverse	ATTGTACTGCAGGTGTACCTTAAAAC	GAPDH_human_reverse	GTGGTCCAGGGGTCTTACTC

Table S7. List of primers used in RT-qPCR
